# Ketogenic Diets and Exercise Performance

**DOI:** 10.3390/nu11102296

**Published:** 2019-09-26

**Authors:** Kristin L. Harvey, Lola E. Holcomb, Stephen C. Kolwicz

**Affiliations:** Heart and Muscle Metabolism Laboratory, Health and Exercise Physiology, Ursinus College, Collegeville, PA 19426, USA; krharvey@ursinus.edu (K.L.H.); loholcomb@ursinus.edu (L.E.H.)

**Keywords:** ketosis, endurance exercise, ketone supplements, obesity, ketone bodies, metabolism

## Abstract

The ketogenic diet (KD) has gained a resurgence in popularity due to its purported reputation for fighting obesity. The KD has also acquired attention as an alternative and/or supplemental method for producing energy in the form of ketone bodies. Recent scientific evidence highlights the KD as a promising strategy to treat obesity, diabetes, and cardiac dysfunction. In addition, studies support ketone body supplements as a potential method to induce ketosis and supply sustainable fuel sources to promote exercise performance. Despite the acceptance in the mainstream media, the KD remains controversial in the medical and scientific communities. Research suggests that the KD or ketone body supplementation may result in unexpected side effects, including altered blood lipid profiles, abnormal glucose homeostasis, increased adiposity, fatigue, and gastrointestinal distress. The purpose of this review article is to provide an overview of ketone body metabolism and a background on the KD and ketone body supplements in the context of obesity and exercise performance. The effectiveness of these dietary or supplementation strategies as a therapy for weight loss or as an ergogenic aid will be discussed. In addition, the recent evidence that indicates ketone body metabolism is a potential target for cardiac dysfunction will be reviewed.

## 1. Introduction

Nutritional intervention and supplementation remain popular strategies for the reduction of body weight and/or for the enhancement of exercise performance. Recently, the ketogenic diet (KD) has emerged as a celebrated dietary plan for the treatment of obesity and diabetes [1]. In addition to being used for weight and body composition management, KDs and/or “keto” supplements are a prominent point of interest within the athletic community for the potential promise as a “superfuel” [2,3]. Although the KD has been used successfully for certain health conditions, questions remain on the long-term impact of the diet on obesity, diabetes, and risk factors for cardiovascular disease (CVD). In addition, the use of the KD as a performance-enhancing substance is still a subject of debate.

KDs are low-carbohydrate, high fat, moderate protein diets that typically supply approximately 80% of calories from fat, 15% calories from protein, and 5% calories from carbohydrates [4,5]. For almost 100 years, the diet has been used for the treatment of epilepsy, but has been reintroduced to the public over last few years. In theory, the high fat content combined with the low carbohydrate intake is purported to stimulate fat oxidation and promote fat loss. Although the diet itself is controversial [4,6,7], KD consumption induces a physiological metabolic state of elevated serum ketone bodies known as “ketosis” in which the cellular oxidation of ketone bodies is enhanced [8]. Recent evidence suggests that increased reliance on ketone body metabolism offers a metabolic advantage in the failing heart and an ergogenic aid for exercise performance [2,3,9,10,11]. These studies, and others, have stimulated interest in the potential health and performance enhancing benefits of KDs. 

The presence of KDs in mainstream and social media, combined with the abundance of “keto” supplements available for purchase, make the diet and supplements extremely attractive for weight loss and athletic performance enhancement. However, the medical community has been reluctant to offer endorsement, due to the potentially negative side effects inherent in the consumption of a diet extremely high in fats with minimal carbohydrates. Moreover, the inconsistent findings prominent in the research literature offer little support from the scientific community. This review will describe ketone body metabolism, define the KD, and summarize the available literature on the potential benefits of KDs and keto-supplements for exercise performance.

## 2. Overview of Ketone Body Metabolism 

Ketone bodies were once considered consequences of aberrant metabolism, produced by the incomplete oxidation of fat due to insufficient carbohydrate availability [12]. This perception led to the labeling of ketone bodies as “metabolic garbage” and “villains of metabolism” [13]. However, ketone bodies are present in the circulation during fed and fasted conditions, although serum concentrations are generally low under basal conditions, typically 0.1 to 0.4 mM in humans and rodents. Ketone body concentration is elevated during periods of nutrient deprivation or low carbohydrate availability, such as fasting/starvation [14], exercise [15], or diabetes [16,17], encompassing both physiological and pathological conditions. In humans, serum ketone body levels are 1–4 mM after a short-term fast (2–3 days), and elevate to 7–9 mM with prolonged fasting (17–24 days) [5,18]. Post-exercise, humans can achieve serum ketone body concentrations of 1–2 mM [19,20]. With low carbohydrate or KDs, serum ketone body levels can increase to above 5 mM [19]. Our published and unpublished data in mice, and those of other research groups, show values of serum ketone bodies comparable to humans: 0.2–0.4 mM (basal); 0.6–0.8 mM (6-hour fasting); >3.0 mM (prolonged fasting); 0.8–1.2 mM (post-exercise); ~1.0 mM (KD) [14,15,21,22,23]. 

**Figure 1:**
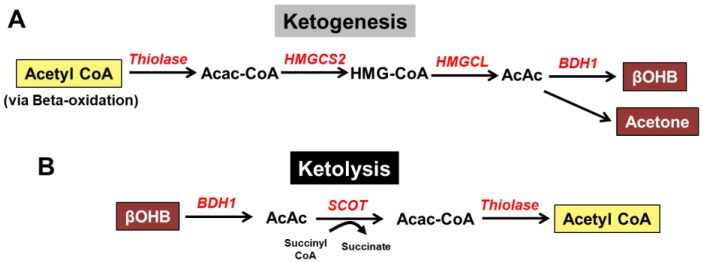
Overview of ketone body metabolism. (**A**) The formation of ketone bodies (βOHB, AcAc, and acetone) from acetyl CoA (via beta-oxidation) occurs in the liver via ketogenesis; (**B**) the breakdown of ketone bodies (βOHB) in the cells via ketolysis yields acetyl CoA. AcAc, acetoacetate; AcAc CoA, acetoacetyl CoA; βOHB, beta-hydroxybutyrate; BDH1, mitochondrial beta-hydroxybutyrate dehydrogenase; HMGCS2, 3-hydroxy-3-methylglutaryl-CoA synthase; HMGCL, HMGC-CoA lyase; SCOT, succinyl-CoA:3-oxoacid-CoA transferase.

Ketone bodies are short-chained, four-carbon molecules synthesized in liver mitochondria through a process called “ketogenesis.” The ketogenic process (Figure 1A) requires acetyl-CoA, generated via the beta-oxidation of fatty acids, and continues with the aid of several enzymes, including mitochondrial acetyl-CoA acetyltransferase 1 (also known as thiolase), 3-hydroxy-3-methylglutaryl-CoA synthase (HMGCS2), HMGC-CoA lyase, and mitochondrial beta-hydroxybutyrate dehydrogenase (BDH1). This process ultimately results in the production of the primary ketone bodies released into the bloodstream: acetoacetate (AcAc) or β-hydroxybutyrate (β-OHB). The ketone body with the highest concentration within the blood is β-OHB; therefore, the breakdown or “ketolysis” pathway generally begins with β-OHB (Figure 1B). Once in the circulation, β-OHB may enter the heart or skeletal muscle cell via monocarboxylate transporters, MCT1 and MCT2 [24]; however, since β-OHB is a short-chain fatty acid, simple diffusion is also possible. β-OHB is then rapidly oxidized into AcAc via BDH1, and subsequently, converted to acetyl CoA via succinyl-CoA:3-oxoacid-CoA transferase (SCOT), and thiolase, for entry into the tricarboxylic acid (TCA) cycle.

Ketone bodies are suggested to be a more energy efficient substrate than glucose or fatty acids [5,25]. However, energy efficiency may be interpreted in multiple ways. In terms of ATP production efficiency, fatty acids yield ~6.7 ATP per carbon atom, compared to ~5.2 for glucose and ~5.4 for ketone bodies [26,27]. For many researchers, energy efficiency refers to the ATP yield per oxygen atom (i.e., P/O ratio). The P/O ratio for fatty acids is ~2.33, while glucose’s is 2.58 and ketone bodies’ is 2.50 [26,27]. For some researchers, cardiac (or muscle) efficiency is a critical measure, which determines the ratio of mechanical work to oxygen consumed. Determination of these values requires complex experimental designs and data collection techniques, which are commonly performed in isolated, perfused heart preparations. In addition, the intricate interactions of certain biochemical pathways may lead to increased energetic costs and/or losses, particularly for fatty acids [5]. Ketone bodies combined with glucose were shown to elicit a higher cardiac efficiency relative to glucose alone [28,29]. Although ketone bodies increased energy production, particularly in hypertrophied hearts, a significant improvement in cardiac efficiency was not achieved [30]. Whether provisions of ketone bodies, glucose, and/or fatty acids lead to a significant alteration in cardiac or skeletal muscle efficiency in healthy or diseased states remains unclear.

Ketosis is considered a physiological condition characterized by an acute state of elevated serum ketone body concentrations, from 0.5 to 3.0 mM. As noted above, such ranges can be achieved via short-term fasting, exercise, or low-carbohydrate diets. Furthermore, ketosis can be classified into “physiological ketosis,” which occurs during fasting or exercise, and “nutritional ketosis,” induced by nutritional or supplementation strategies. The metabolic state of ketosis should not be confused with ketoacidosis. In contrast to ketosis, ketoacidosis is a pathological condition with elevated serum ketone levels (3.8–25 mM) and decreased arterial pH values (7.30 to 7.20) that may be seen in diabetics [31,32,33]. Therefore, these specific terms should be used for clarity when evaluating the systemic effects of elevated ketone body concentrations.

## 3. Ketogenic Diets and Weight Loss

The prevalence of obesity in the United States remains a significant public health issue, as nearly one-third of men and women are classified as overweight or obese [34]. This increase in body weight and adipose tissue mass increases the risk of developing hypertension [35], type II diabetes [36], and CVD [37]. Behavioral modifications, specifically dietary strategies and physical activity/exercise, are recommended to combat obesity. Over the years, numerous “fad diets,” such as the Atkins or South Beach diets, have been popularized for weight loss with varying levels of patient outcomes, scientific evidence, and support from the medical community [38]. More recently, the KD has been proposed as a strategy for combatting obesity, since it induces rapid weight loss. The lack of carbohydrate availability in the diet is proposed to induce fatty acid mobilization from adipose tissue as a way to supply energy to the body via ketone bodies, resulting in an efficient method of promoting weight loss [4].

Although the precise composition of the KD varies throughout the literature, the diet is typically defined as a low carbohydrate diet that contains adequate protein (15–20%) and high caloric intake from fat. The original KD was developed by physician-researchers, Woodyatt and Wilder, at the Mayo Clinic in the early 1920s, as a treatment for diabetics and epileptic children [39,40]. The dietary regimen called for 1 gram per kg of body weight of protein, 10–15 grams of carbohydrates per day, and the balance of the intake in the form of fats, resulting in an approximate fat to protein plus carbohydrate ratio of 4:1. That dietary composition is the classical Peterman KD, named after the physician who reported the formulation [41]. The KD was a frequent treatment for epilepsy in children until the introduction of antiepileptic drugs in the late 1930s, but remained prominent in medical textbooks until the 1980s [42]. The KD reemerged in the late 1990s as a treatment for intractable epilepsy or refractory seizures [42], and remains a viable therapy in instances where pharmacological intervention is not sufficient. In the last 20 years, the KD has been effective in the treatment of patients with inherited metabolic disorders, such as glucose transporter type 1 (GLUT1) deficiency, pyruvate dehydrogenase deficiency, and glycogen storage diseases [43].

Over the last decade, research on the effectiveness of the KD in a variety of diseases and conditions has increased significantly. Human studies typically use low-carbohydrate (LC), which is not necessarily a true KD, or very low-carbohydrate ketogenic diets (VLCKD), in which carbohydrate intake can range between 20–50 grams per day or less than 10% of calories, respectively [44,45]. In some studies, the term Atkins diet is also used [46,47]. In other studies, the LC diets are more aligned with reduced carbohydrate diets (intakes of 35–45% calories per day) [48,49]. Thus, the precise characteristics of the dietary intervention are important in the ultimate interpretation of the results.

Several human studies evaluated the benefits of LC or KD diets on weight loss. In a one-year study of 160 overweight and obese individuals, the Atkins diet lowered body weight, cholesterol, and insulin to the same degree as low-fat, caloric restriction, or reduced carbohydrate (40% of calories) diets [46]. A similar observation was made in 90 patients with increased risks of CVD and type 2 diabetes after 6 months of dietary intervention [47]. A 12-week LC diet in adolescents led to significant reductions in body weight and LDL cholesterol compared to a low-fat diet [45]. Conversely, LC diets showed similar weight loss compared to low-fat diets in obese patients after 2 years [44,50]. However, the LC diet was met with an improvement in blood lipid profiles and other CVD risk factors [44,50]. A reduced carbohydrate diet (35–45% caloric intake) showed similar weight loss compared to a reduced fat diet (20% calories) in approximately 400 and 800 overweight adults after 2 years of treatment [48,49]. In a meta-analysis study encompassing ~1600 patients, a VLCKD diet achieved greater weight loss, reduced diastolic blood pressure, lowered serum triglyceride levels, and elevated HDL levels compared to a low-fat diet at 12 and 24 months [51]. Unfortunately, LDL levels were significantly higher in the VLCKD patients [51]. In total, a reduction and restriction of carbohydrate intake appears to be sufficient to promote weight loss. However, whether this dietary strategy is more effective than other methods is not clear. It should be noted that the above studies did not evaluate serum ketone body levels, so whether the weight loss is associated with ketosis or a ketogenic effect is not known.

In the aforementioned human studies, a major limitation is patient adherence to the assigned dietary intervention. Therefore, animal studies, particularly in rodent models, may provide better insight. Some studies in mice utilize diets that are more reflective of a true KD, in which fat represents 90–95% of the total calories, protein 5–10%, and carbohydrates ~1% [14,21,52,53]. When C57BL6/J mice are fed a KD for 5 to 8 weeks, mild weight loss (~10%) occurs, particularly in the first 1–3 weeks [14,21]. In addition, KD-fed mice gain significantly less weight than mice fed a high fat diet (HFD, 60% of total calories) [21]. Although a 5-week treatment of KD in mice being fed a HFD for 12 weeks reduces body weight [21], KDs in ob/ob mice are not effective at reducing obesity [52]. Moreover, long-term treatment (22 weeks) of mice with KD does not result in body weight changes and may lead to glucose intolerance and insulin resistance [53]. These studies support the KD as a weight loss strategy in mice, particularly in the short-term. However, long-term consumption of the KD, especially with an extremely high fat content and reduced protein, may result in unexpected consequences. 

## 4. Ketogenic Diets and Exercise Performance

### 4.1. Overview of Metabolism During Exercise

Actively-contracting muscles receive the contributions of three major energy pathways, which are influenced by the time and intensity of the exercise [54]. The phosphocreatine (PCr) to ATP reaction, regulated by creatine kinase (e.g., the phosphagen system), is essential in resynthesizing ATP during immediate, high intensity work, and is a dominant system during the initial seconds of exercise. In moderate to high-intensity exercise sessions, lasting up to ~90 s, the short-term lactic acid system is a major contributor. During this interval, ATP resynthesis is primarily met by glycogen-dependent glycolysis [55]. In moderately intense, long-duration exercise, the long-term aerobic system supplies metabolic substrates to support oxidative metabolism. Oxygen demands and oxygen uptake determine the contribution of the above energy systems during the metabolic response to exercise. During the initial moments of exercise, a large increase in oxygen uptake is required to match the energetic demands of the contracting muscle cells. However, a mismatch between the metabolic demands and oxygen uptake exists for several seconds to several minutes, called the “oxygen deficit” [56]. During the oxygen deficit, the phosphagen system and lactic acid system are the major supporters of ATP resynthesis. Once oxygen uptake and oxygen demand are in balance, oxidative phosphorylation via the aerobic system becomes the dominant pathway to maintain ATP regeneration.

**Figure 2:**
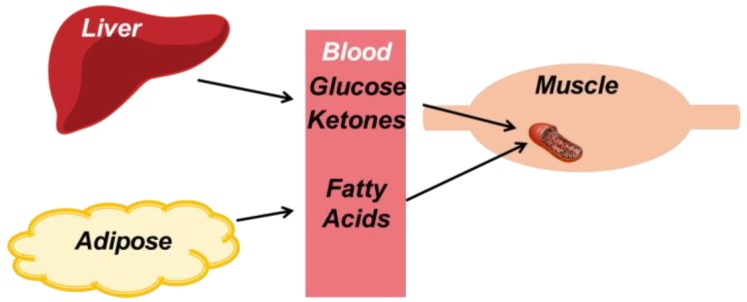
The exogenous supply of substrates during exercise. During exercise, skeletal muscle requires a constant supply of exogenous substrates to fuel contraction. The liver provides glucose and ketone bodies via gluconeogenesis and ketogenesis, respectively. Adipose tissue lipolysis maintains serum fatty acid concentrations.

Once steady-state aerobic metabolism is reached, a steady supply of exogenous substrates are needed to maintain exercise. As shown in Figure 2, these exogenous substrates are supplied by the liver and adipose tissue. During aerobic exercise, the liver has the primary role of maintaining blood glucose levels via glycogenolysis, and to a smaller degree, gluconeogenesis. In addition, the liver can produce ketone bodies from elevated serum concentrations of fatty acids. A sustained rise in serum fatty acids occurs due to the lipolysis of adipose tissue, activated by beta-adrenergic stimulation [57]. Through these coordinated efforts of the liver and adipose tissue, a sufficient supply of substrates, namely, glucose, ketone bodies, and fatty acids, fuels the contraction of cardiac and skeletal muscle. Cardiac muscle has an added benefit, as it demonstrates an increased capacity to utilize lactate produced by the skeletal muscle during higher workloads [58].

### 4.2. The Effects on Aerobic Endurance Exercise 

The contribution of fatty acids to oxidative metabolism varies with exercise intensity and duration [59]. During low-to-moderate intensity exercise, the oxidation of exogenous fatty acids is a significant source of energy. During exercise of a moderate intensity, the contribution of fatty acids to oxidative metabolism increases, as the duration of the exercise bout is prolonged. In that regard, strategies that promote the availability of fatty acids may be critical to optimizing endurance exercise performance. The KD may be advantageous, especially for aerobic endurance exercise, by promoting fat usage, rather than carbohydrates, for fuel. Fat from adipose tissue is considered a steady supply of energy, while endogenous carbohydrate stores from glycogen in the skeletal muscle and the liver are finite. Elevated ketone bodies, resulting from the KD, may provide an alternative or supplemental fuel source to sustain endurance exercise.

There are numerous studies over the past decade that examined the effect of low carbohydrate (LC) or KD (LC/KD) diets on endurance exercise performance in humans [20,60,61,62,63,64,65,66,67,68,69]. A vast majority of the studies focused on endurance-trained individuals, and included primarily male athletes. The diets utilized in the studies varied with the average caloric intake percentage from fat at 73% (range 63–80%); carbohydrates 7% (3.5–15%); and protein 20% (15–29%). Treatment times varied from as little as 3 weeks [60,66] up to 20 months [20]. Serum ketone body concentrations (mostly βOHB) reportedly increased anywhere from 0.5mM to 1.2mM, and did not appear to relate to the fat composition or treatment time of the diets. Most of these studies reported significant decreases in body weight or fat mass [60,64,65,66,67,69]. Therefore, LC/KD diets appear to be an effective dietary strategy to induce weight loss and improve body composition in trained athletes.

However, despite the positive changes in body and fat mass, LC/KD diets are not effective in producing significant improvements in exercise performance, despite significant decreases in respiratory exchange ratio (RER), representing an increase in fatty acid oxidation (FAO). LC/KD did not significantly alter total time to exhaustion (TTE) [62,67], maximal oxygen uptake (VO_2_max) [61,62,63,64,67,69], or endurance cycling performance [65]. In contrast, the consumption of a LC/KD for 3 weeks, combined with exercise training, impaired the training adaptations of elite race walkers by elevating oxygen consumption rates during activity [60]. In 30-year-old endurance trained males fed a LC/KD for 1 month, TTE was reduced at 70% intensity, despite no change at 60% intensity [67]. Two studies that included a population of females presented interesting results [63,69]. In a small study of endurance athletes, 90% comprised of females, TTE was significantly decreased after 10 weeks of LC/KD [69]. Similarly, LC/KD fed females from a recreational-trained Cross-Fit cohort experienced a non-significant 5% decrease in VO_2_max, while males were unaffected by the diet [63]. These studies clearly show that LC/KD in trained individuals offers no enhancement in exercise performance, and may lead to decreased performance, particularly in females. 

Studies on the effects of LC/KD on exercise performance in overweight/obese individuals are limited and revealed varying results [70,71,72,73]. An early study suggested that moderately obese individuals (primarily female) following a reduced carbohydrate (CHO) diet (45% calories) lost significant body weight and fat mass, and had improved endurance times during moderate exercise intensity [72]. Although obese females consuming a diet of 33% CHO combined with exercise training experienced 20% greater weight loss, the improvement in TTE was similar to high CHO diet [72]. A LC/KD for middle-aged, obese adults for 52 weeks led to a greater decrease in body weight and fat mass, compared to a low calorie or mixed diet, but did not result in improved exercise performance [74]. In overweight/obese adults, a LC/KD diet led to significant weight loss only in males, with no significant change in TTE or VO_2_max in males or females compared to a low fat diet [70]. However, a 2-week LC/KD diet in overweight adults did not lead to weight loss, but increased fatigue and perceived effort [73]. It should be noted that the keto-adaptation period is suggested to be 2–4 weeks [75], so results of very short dietary interventions should be interpreted with caution. Although LC/KDs appear effective in the management of body weight and fat mass in overweight and obese individuals, the effects on exercise performance remain unclear and may depend on the degree of carbohydrate restriction and length of the dietary intervention.

Studies in rodents fed a LC/KD diet may provide some additional mechanistic insight into the effects of the LC/KD on exercise performance, particularly since the diet can be well controlled and the capability of performing bio-molecular measures is not limited. The composition of the LC/KD fed to rodents typically ranges from 70–78% fat with 1–5% CHO and 9–20% protein [76,77,78,79,80]. In C57BL6/J male mice, 8 weeks of KD improved exercise treadmill times and molecular markers of recovery [77,78]. However, 4 weeks of KD fed to female C57BL6 mice decreased aerobic capacity [80]. In Sprague Dawley rats, voluntary running distance was not different during 6 weeks of KD [76]; however, run time to exhaustion on a treadmill was improved after 1 or 5 weeks of KD [79], compared to chow-fed controls. In addition to variable reports in exercise performance, some potential negative side effects, including increased adipose tissue mass [78,80], decreased muscle glycogen content [79], increased serum triglycerides [80], and decreased cardiac function [80], were noted. However, KDs may decrease mortality [81,82], improve memory [81], and increase muscle citrate synthase [82] in aged mice. Perhaps additional studies in animal models are necessary to help derive definitive conclusions.

### 4.3. Its Effects on Anaerobic Exercise 

Anaerobic exercise is a high intensity, low duration exercise that lasts less than 2 min. Energy demands are met by the phosphagen system and lactic acid system, which are highly dependent upon skeletal muscle glycogen. During anaerobic exercise, high contractile forces occur within the muscle, and muscle fibers become damaged. In addition to the replenishment of carbohydrates during the recovery period, adequate consumption of essential amino acids is important to support the protein synthesis necessary to repair and rebuild the muscle. In this regard, LC/KDs typically provide sufficient protein intake (~15% of daily calories) to avoid amino acid deficiency. However, due to the low carbohydrate intake, the increased reliance of amino acids toward gluconeogenesis and the impairment of glycogen-store restoration may adversely affect anaerobic performance.

Several studies evaluated the effects of LC/KDs on anaerobic performance, primarily assessing power or strength parameters, in various populations, including endurances athletes [65], Cross-Fit participants [83,84], gymnasts [85], and powerlifters [86]. Dietary interventions ranged from 6 weeks up to 12 weeks, and included normal training regimens typical of the populations studied. In general, consumption of the LC/KD did not result in strength [64,84,85,86] or power [83,84] measures that were significantly different from the control groups. One study reported a significant increase in relative power, but not absolute power, which was due to the decreased body weight experienced by the subjects [65]. In some studies, significant decreases in skeletal muscle thickness [64] or lean body mass [83] were noted. Moreover, muscle hypertrophy from resistance training may be blunted with the LC/KD [87]. These studies demonstrate that the LC/KD diet is not an effective strategy to increase anaerobic performance in trained individuals or athletes, and it has the potential to negate the expected increases in lean body mass from anaerobic training.

## 5. Ketone Body Supplementation

As LC/KDs require high fat consumption and present difficulty with long-term adherence, alternative methods for targeting ketosis as a potential intervention for weight loss or as an ergogenic aid are required. In support of these, several studies examined the benefits of ketone body supplements on exercise performance [2,88,89,90,91,92]. Ketone body supplements are commercially available and commonly present in the form of ketone salts (KS) or ketone esters (KE). Additionally, medium chain triglycerides (MCT) are sometimes used to induce ketosis [89] or are combined with KS to maximize the ketotic response [93].

The formulation of KS may include βOHB or 1,3-butandeniol (BD), bound to sodium, potassium, or calcium. There are a few potential concerns with consuming KS. First, βOHB in the salt form could include both D and L enantiomers of βOHB. Since D-βOHB is the biologically active form, approximately 50% of elevated serum βOHB levels are due to the presence of the non-metabolizable L-βOHB that must be excreted via the urinary system [94]. As such, KS appears less effective at elevating serum βOHB comparatively [93,94]. Second, BD is a compound that must be converted to βOHB in the liver via dehydrogenase enzymes [95], which may result in delays in increased serum βOHB concentration [93]. Finally, the increased consumption of mineral salts, particularly sodium, may adversely affect blood pressure.

In the last few years, most research studies utilized KE, which appears to be the most effective method to cause immediate and sustained increases in serum ketone bodies. There are several formulations of KE supplements, but the most identifiable is the (R)-3-hydroxybutyl (R)-3-hydroxybutyrate ketone monoester, which converts to in D-βOHB and BD upon ingestion [96]. This particular KE, when taken in combination with CHO, results in a 2% increase in exercise performance in trained cyclists [2]. However, not all KE supplements increase exercise performance [90,91,92], calling into question whether the precise formulation of the KE is essential or whether an additional substrate like CHO is required. Of note, the available studies focused on exercise performance in trained endurance athletes, so whether supplementation in recreational athletes or fitness enthusiasts is appropriate is not known.

## 6. Ketone Body Metabolism and the Heart

The high-energy demand of the heart requires a steady supply of carbon-based substrates in order to regenerate the ATP necessary to maintain contraction. As such, the heart is capable of utilizing all exogenous substrates (fatty acids, glucose, lactate, ketone bodies, and amino acids), as well as endogenous substrates (triglycerides and glycogen). Past research established that fatty acids contribute 60–80% of the fuel supply in the normal, healthy heart, whereas glucose contributes 10–20% [97,98,99,100]. Ketone body oxidation was generally thought to play a relatively minor role, contributing ~10–20% in the healthy myocardium [23,100], with a relatively unknown role in the diseased heart. Since LC/KDs are in stark opposition to current guidelines published by the American College of Cardiology/American Heart Association (ACC/AHA) [101], there exists a major concern on whether the KD will promote the development and/or progression of CVD.

Cardio-metabolic derangements in various diseased states are well established in the literature. In the hypertrophied and failing heart, past research identified significant decreases in fatty acid oxidation (FAO) that is matched by a marked increase in glycolysis [99,101,102]. In the obese and diabetic heart, a mismatch between the uptake and oxidation of elevated exogenous fatty acids contributes to the development of “cardiac lipotoxicity” [97]. In general, both ketone body and amino acid metabolism have often been overlooked or deemed insignificant during cardiac disease until recently [9,10,11,30,103]. Recent work identified that ketone body metabolism is upregulated in the hypertrophied and failing heart [9,10,11], which might serve as an alternative fuel source to sustain myocardial function. Despite the recent provocative findings [9,10,11,104], the role of ketone body metabolism and/or KDs on cardiac metabolism and function, under both physiological and pathological conditions, remains relatively unexplored.

A recent study demonstrated that the hypertrophied and failing heart upregulates BDH1 expression and increases ketone body oxidation by 25% [9]. Furthermore, cardiac-specific deletion of BDH1 worsens cardiac function and remodeling in a mouse heart failure model [11]. Interestingly, a 4-week administration of a KD (80% fat, 20% protein and ~0% carbohydrates) improves cardiac remodeling in heart failure [11]. These findings suggest that the targeting of ketone body metabolism, particularly via a KD, might be a potential therapeutic treatment for cardiac dysfunction. However, a provision of ketone bodies to the hypertrophied heart may not improve cardiac efficiency [30] and controversy exists on whether KDs improve or exacerbate recovery from cardiac ischemia [105,106,107]. Moreover, in diabetic rats, long-term KD treatment may worsen diabetic cardiomyopathy [103]. Therefore, additional studies focusing on the alterations in cardiac metabolism and function elicited by long-term treatment of LC/KDs are needed in order to identify the safety and efficacy of this dietary strategy in cardiac disease.

## 7. Potential Side Effects of Ketogenic Diets

In addition to the documented changes in body weight in humans [108,109] and animals [14,21], LC/KDs are suggested as a promising therapy for various neurological disorders [110,111,112], metabolic disorders (Type 2 diabetes) [113,114], risk factors for CVD [115,116], and certain types of cancer [117,118]. The literature also suggests that an LC/KD extends lifespan in mice [81,119] and augments memory in mice and humans [81,120]. The KD may also be effective at targeting mitochondria, including mitochondrial biogenesis, mitochondrial function, and mitochondrial DNA mutations [121,122,123,124]. Although difficulty remains in reconciling the effects of elevated fatty acid versus elevated ketone body metabolism, future mechanistic studies may provide greater insight, especially with the use of supplementation strategies.

Despite the potential benefits of LC/KD strategies, various concerns remain. Studies examining the effect of KD on liver steatosis [125,126], glucose homeostasis [126,127], and dyslipidemia [53,128] remain controversial. Recent evidence expresses potential concerns with the LC/KD compromising bone health in rodents as several studies reported decreases in bone mineral content and bone density [129,130,131]. In a transgenic mouse model of neurodegeneration, KD caused acceleration of the neurodegenerative process and induced mitochondrial dysfunction, despite the promotion of mitochondrial biogenesis [132]. Furthermore, a short-term treatment of a LC/KD (60% fat, 15% CHO) failed to improve memory and learning in apparently healthy humans [133]. Depending on the dietary carbohydrate sources, LC/KD may also lead to micronutrient deficiencies [134]. Certainly, additional longer-term studies are required to address the safety and efficacy of LC/KDs in both healthy and diseased states.

Epileptic children appear to be particularly vulnerable to potential negative side effects of the KD. Decreases in bone mineral content and bone loss have been documented [135,136]. In addition, the concern of kidney stones exists in approximately 6% of children with intractable epilepsy on the KD, which may be reduced with administration of potassium citrate [137,138]. Interestingly, recent studies have identified significant reductions in the hormone ghrelin, which may be responsible for low growth rates in epileptic children [139,140]. Future investigations should focus on the characterization of hormonal changes that may occur with KD consumption in adults. 

## 8. Summary and Conclusions

For endurance athletes, the literature supports LC/KDs as an effective strategy to reduce body weight and fat mass, particularly in the period of 3–12 weeks. Limited studies demonstrate a significant improvement in exercise performance at submaximal (~60%) intensities. However, exercise performance at higher intensities may actually be impaired. For athletes concerned with anaerobic power and strength, short-term consumption of LC/KDs does not negatively affect these performance parameters but may lead to unwelcomed decreases in lean body mass or blunted skeletal muscle hypertrophy. Therefore, the literature does not support the use of LC/KD as an effective dietary strategy to increase athletic performance.

Ketone body supplements, including KS and KE, are commercially available and gaining popularity in the exercise community. However, since supplements are not evaluated or approved by the Food and Drug Administration (FDA), consumers must pay careful attention to the components of the supplements. Compared to KS, KE supplements appear to be more effective at inducing ketosis; however, there are limited studies demonstrating improvements in the exercise performance of trained athletes. Moreover, the benefits of KE supplementation in non-athletes is unknown.

Although recent research findings lend support to targeting ketone body metabolism for the treatment of cardiac dysfunction, obesity, diabetes, and exercise performance, further research is needed before dietary interventions or supplementation is implemented. Individuals who do decide to use LC/KDs or ketone body supplements should do so with caution.
